# 

**Published:** 1877-08

**Authors:** 


### Books and Pamphlets Received.

Surgical Observations, with Cases and Operation. By J. Mason Warren, M. D. Published by Ticknor and Fields, Boston, 1867. For sale by A. Williams & Co., Boston, 1877.

Half-Yearly Compendium of Medical Science.

The Popular Science Monthly, and the Supplement for July and August.

Reply to Dr. J. Marion Sims. By Drs. Peaslee, Emmet & Thomas.

Transactions of the Medical Society of West Virginia.

Thirty-Fourth Annual Report of the Managers of the State Lunatic Asylum, Utica, N. Y., for 1876.

Transactions of Medical and Chirurgical Faculty of Maryland, April, 1877.

Recuring Sarcomatous Tumor of the Orbit in a Child. By Thomas Hay, M. D. Philadelphia: Lindsay & Blakiston, 1877.

Notes on the Epidemiology of Ohio. By J. C. Minor, M. D. Reprint from Cincinnati Lancet and Observer, 1877.

Analysis of Seven Hundred and Seventy-four Cases of Skin Disease. By L. Duncan Bulkley, A. M., M. D. Reprint from New York Medical Journal, June, 1877.

Medical Organizations and their Value. By Wm. B. Atkinson, M. D., 1877.

Annual Announcements for 1877 and 1878, of Bellevue Hospital Medical College; Medical Department of University of Michigan; Hospital College, Medical Department of Central University, Louisville, Ky.; and Medical Department of University of New York.

